# Measuring cortisol in Cushing syndrome: diagnosis, monitoring, and cortisol circadian rhythm improvement

**DOI:** 10.1210/clinem/dgag095

**Published:** 2026-03-14

**Authors:** Oksana Hamidi, Matthieu St-Jean, André Lacroix, Irina Bancos

**Affiliations:** Division of Endocrinology, University of Texas Southwestern Medical Center, Dallas, TX 75390-8857, USA; Division of Endocrinology, Department of Medicine, Centre hospitalier de l’Université de Sherbrooke, Sherbrooke, QC J1H 5H3, Canada; Division of Endocrinology, Department of Medicine, Centre hospitalier de l’Université de Montréal, Montreal, QC H2W 1T8, Canada; Division of Endocrinology, Diabetes, Metabolism and Nutrition, Mayo Clinic, Rochester, MN 55905, USA; Department of Laboratory Medicine and Pathology, Mayo Clinic, Rochester, MN 55905, USA

**Keywords:** cortisol circadian rhythm, hypercortisolism, Cushing syndrome, mild autonomous cortisol secretion, treatment monitoring, cortisone

## Abstract

Endogenous Cushing syndrome (CS) is a rare disorder resulting from chronic exposure to excessive concentrations of cortisol. It is likely underdiagnosed because many clinical signs and symptoms are non-specific and overlap with those of common conditions. Furthermore, biochemical testing to diagnose CS can be complex and challenging, especially in milder cases. CS is characterized by excessive daily cortisol production, but it is also associated with a disrupted circadian rhythm of cortisol secretion. Traditional cortisol monitoring techniques rely on single-time-point measurements or a cumulation of measurements, which are unable to capture the complete daily rhythm of cortisol fluctuations. Currently, the utility of assessing individual patients’ cortisol circadian rhythm during diagnosis and treatment of CS is not well characterized. In this review, we will discuss how cortisol is measured in clinical practice and the potential benefit of measurement and normalization of the cortisol circadian rhythm.

The hypothalamic pituitary adrenal (HPA) axis regulates the circadian rhythm of cortisol secretion by coordinating the release of cortisol secretagogues (1-4). The hypothalamus secretes corticotropin-releasing hormone (CRH) and vasopressin, which stimulate the pituitary gland to release adrenocorticotropic hormone (ACTH) (5-8). In turn, ACTH stimulates the adrenal glands to produce and secrete cortisol (9). The increase in serum cortisol then acts via negative feedback loop to inhibit further production of CRH, vasopressin, and ACTH (Fig. 1) (10). ACTH and cortisol are normally released in a pulsatile fashion, with high serum concentrations in the early morning that decline throughout the day reaching nadir cortisol concentrations around midnight in humans with usual sleep–wake cycles (11, 14-16). The ACTH–cortisol circadian rhythm is established during the first year of life and is essential for many physiological functions, including the regulation of energy metabolism, immune function, and stress response (17-20).

**Figure 1:**
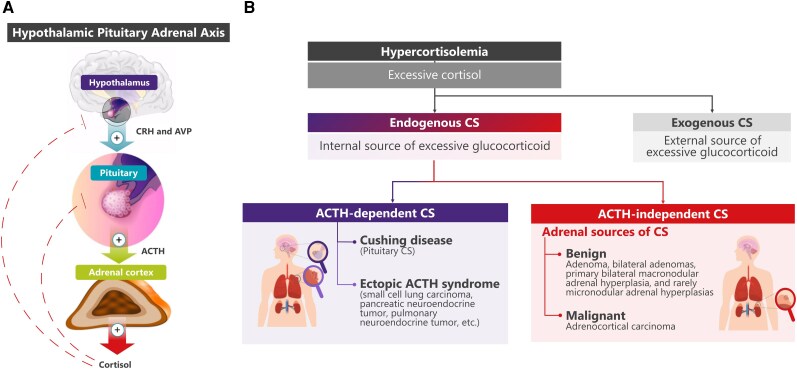
Hypothalamic pituitary adrenal axis and Cushing syndrome etiologies (5-13). The hypothalamus secretes CRH and vasopressin to stimulate pituitary ACTH release, which in turn stimulates adrenal cortisol production; cortisol exerts negative feedback on CRH, vasopressin, and ACTH (panel A). CS is classified as exogenous (due to glucocorticoid administration) or endogenous (Panel B). Endogenous CS is further subdivided into ACTH-dependent (pituitary or ectopic ACTH production) and ACTH-independent (primary adrenal unilateral or bilateral lesions). Abbreviations: ACTH, adrenocorticotropic hormone; AVP, vasopressin; CRH, corticotropin-releasing hormone; CS, Cushing syndrome.

Cushing syndrome (CS) is a disorder resulting from chronic exposure to excessive levels of cortisol (11, 21). Depending on the cause, CS can be classified as exogenous (due to administration of synthetic glucocorticoids via diverse routes) or endogenous (a rare set of disorders in which the body produces excess cortisol) (11, 21, 22). This manuscript focuses on endogenous CS, which can be further subdivided as ACTH-dependent (excess ACTH produced from a pituitary or ectopic tumor source) or ACTH-independent (excess cortisol produced from a primary adrenal unilateral tumor or bilateral lesions) (Fig. 1) (11, 12). This leads to inappropriately elevated circulating levels of cortisol throughout the day and night, disrupting the normal circadian rhythm of cortisol secretion (23, 24). The loss of circadian rhythm with the absence of a late-night cortisol nadir is a hallmark biochemical abnormality in patients with CS (11, 25, 26). The loss of cortisol circadian rhythm contributes to multiple complications and ultimately increases morbidity and mortality (27, 28). In addition to circadian rhythmicity, cortisol secretion in healthy individuals exhibits ultradian pulsatility, defined as recurring fluctuations occurring at intervals of less than 24 hours, superimposed on the circadian pattern (29). In patients with CS, cortisol excess is associated with disruption of both circadian and ultradian rhythmicity, rather than solely increased cortisol concentrations (30). The clinical significance of disrupted ultradian rhythms with respect to disease-related morbidity and mortality remains unclear.

The degree of hypercortisolism is highly variable in patients with CS, which results in a wide range of symptoms and clinical presentations ranging from mild to severe forms (31-34). CS can lead to increased morbidity and mortality, regardless of its etiology. Comorbidities resulting from chronic hypercortisolism can be present before diagnosis and persist after remission, and include metabolic syndrome, cardiovascular disease, thromboembolic events, musculoskeletal damage, neuropsychiatric diseases, sleep impairment, immune disorders, and reproductive and sexual dysfunction (32, 35, 36). The Pituitary Society treatment guidelines recommend the normalization of cortisol levels or action at its receptors to decrease or eliminate the signs and symptoms of CS while also treating CS-associated comorbidities (37, 38).

Currently, there is a lack of practical and effective ways to measure cortisol secretion over the course of 24 hours. Conventional cortisol monitoring methods rely on single-time-point measurements or a cumulation of measurements, which fail to capture the full diurnal pattern of cortisol fluctuations. The introduction of a continuous cortisol monitoring device holds the potential to improve both diagnostic precision and monitoring effectiveness in patients with CS (30). Moreover, the clinical impact of restoring the normal circadian rhythm of cortisol on morbidity and mortality remains poorly understood. However, evidence suggests that restoring cortisol circadian rhythm may improve disease control and alleviate symptoms (39-43).

In patients with CS, there is currently no guidance for monitoring treatment response with respect to the restoration of cortisol circadian rhythm. Although this can be achieved after successful surgery selectively removing the source of excess ACTH or cortisol, normalization of the cortisol circadian rhythm after most medical therapies has rarely been investigated and is not routinely measured to assess treatment response (39-44). This review will focus on the tests available for the measurement of cortisol in clinical practice, the benefit of novel cortisol measurement tools, and the potential impact of restoration of the cortisol circadian rhythm among patients with CS. We aim to underscore the importance of cortisol monitoring and restoring the cortisol circadian rhythm, with the goal of improving diagnosis and clinical management of cortisol-excess disorders.

## Diagnostic algorithm

The clinical presentation of CS varies based on the etiology of the disorder and the severity of hypercortisolism (21, 31-34, 45, 46). In patients with CS, clinical presentation is often recognized due to overt physical manifestations of hypercortisolism including reddish purple striae, plethora, proximal muscle atrophy and weakness, supraclavicular and dorsocervical fat pads, bruising with no obvious trauma, and unexplained osteoporosis (11, 47, 48). However, patients with CS may present with much less severe symptoms that are otherwise common in the general population including obesity, hypertension, diabetes, depression, and menstrual irregularities. The diagnosis of endogenous CS can be challenging due to overlap with common conditions, highly variable clinical features, and challenges in appropriately interpreting biochemical screening and diagnostic tests (11, 48-52). These challenges often lead to delayed diagnosis, contributing to increased morbidity and mortality (48-50, 53).

**Figure 2:**
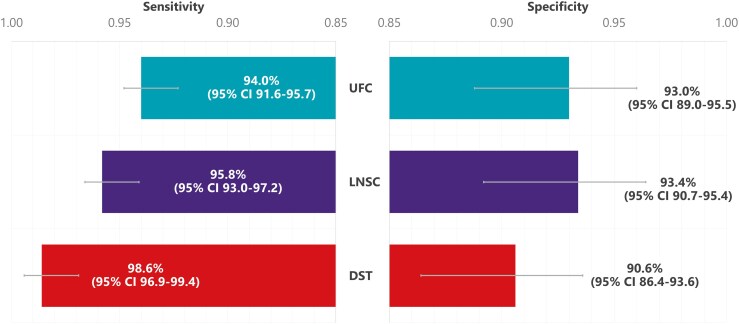
Sensitivity and specificity of first-line screening and diagnostic tests (74). Sensitivity (left panel) and specificity (right panel) with corresponding 95% CIs are shown for 24-hour UFC, LNSC, and DST. This research was originally published in Galm et al 2020 (74). Abbreviations: CI, confidence interval; DST, dexamethasone suppression test; LNSC, late-night salivary cortisol; UFC, urinary free cortisol.

Guidelines recommend first excluding exogenous CS due to glucocorticoid exposure through oral, rectal, inhaled, topical, or injected routes, as well as from other agents with glucocorticoid activity such as high-dose progestogens (eg, megestrol acetate), before performing biochemical screening for hypercortisolism in patients with clinical suspicion of endogenous CS (11, 38). Biochemical screening consists of one of the following tests in cases of low clinical suspicion and 2 or more tests in case of moderate or elevated clinical suspicion: at least 2 measurements of 24-hour urinary free cortisol (UFC), at least 2 measurements of late-night salivary cortisol (LNSC), or 1-mg overnight dexamethasone suppression test (DST) (Table 1). If one or more of the initial test results is abnormal, it is recommended to repeat one or 2 of the screening tests (24-hour UFC, LNSC, or 1-mg DST) and to exclude non-neoplastic sources of hypercortisolism (eg, psychiatric conditions, alcohol use disorder, uncontrolled diabetes, and others). The desmopressin stimulation is a useful tool for distinguishing non-neoplastic hypercortisolism from true CS, with the combined dexamethasone–CRH test as an alternative when available (84, 85). The objective of the screening tests is to evaluate the abnormalities in cortisol secretion that are typically observed in CS: LNSC for elevated nocturnal cortisol due to abnormal cortisol circadian rhythm, overnight 1-mg DST for impaired glucocorticoid feedback, and 24-hour UFC for increased integrated estimate of 24-hour cortisol secretion (11, 38). It is important to keep in mind the sensitivity, specificity, and limitations of each test (Fig. 2). Ideally, the limitations should be considered before ordering the test, taking into account clinical probability of CS. After a biochemical confirmation of CS, plasma ACTH concentration should be measured to distinguish between ACTH-independent (≤10 pg/mL [2.2 pmol/L]) and ACTH-dependent CS (≥20 pg/mL [4.4 pmol/L]) (38, 86, 87). When plasma ACTH levels fall between 10 and 20 pg/mL, this range is considered a “gray zone,” and additional testing is typically required to differentiate ACTH-dependent and ACTH-independent CS (eg, repeat ACTH measurement, desmopressin stimulation test). However, ACTH thresholds are not absolute. Plasma ACTH levels may exceed 20 pg/mL in milder adrenal CS and mild autonomous cortisol secretion (MACS), whereas ACTH levels in pituitary-dependent Cushing disease (CD) are higher and rarely below 30 pg/mL (88, 89). Accordingly, ACTH results should be interpreted in clinical context and alongside other biochemical and imaging findings. Additional tests may include measuring dehydroepiandrosterone sulfate, with low concentrations suggestive of ACTH-independent hypercortisolism and normal-elevated concentrations suggestive of ACTH-dependent hypercortisolism (33). Further imaging and tests are then needed, based on the suspected etiology, to determine the specific subtype of CS (CD, adrenal CS, or ectopic CS) to appropriately and efficiently treat patients (38).

**Table 1:** **First-line screening and diagnostic tests for Cushing syndrome (** 11, 38, 51, 54-83**)**

Test	Measurement	Utility	Strengths	Limitations
24-hour urinary free cortisol (UFC)	Integrated estimate of 24-hour cortisol secretion	Diagnosis Treatment monitoring	Unaffected by CBG Useful for overt CS	Collection error Less sensitive for mild cases Affected by sex, BMI, age, urine volume, GFR, sodium False positives: non-neoplastic hypercortisolism
Late-night salivary cortisol (LNSC)	Detection of the loss of bedtime nadir	Diagnosis Recurrence monitoring Treatment monitoring	Non-invasive Unaffected by CBG High sensitivity and specificity	Unsuitable for disrupted sleep patterns Less sensitive for mild cases False positives: non-neoplastic hypercortisolism, smoking/tobacco users
1-mg Overnight dexamethasone suppression test (DST)	Assessment of glucocorticoid feedback response	Diagnosis Recurrence monitoring	High sensitivity notably for mild cortisol secretion in adrenal incidentalomas	False positives: non-neoplastic hypercortisolism, dexamethasone omission, non-compliance, contraceptives, pregnancy, renal impairment, drug interactions

The table summarizes the measurement, utility, strengths, and limitations of 24-hour UFC, LNSC, and the 1-mg overnight DST.

Abbreviations: BMI, body mass index; CS, Cushing syndrome, DST, dexamethasone suppression test; GFR, glomerular filtration rate; LNSC, late-night salivary cortisol; UFC, urinary free cortisol.

### UFC

Testing for increased bioavailable cortisol with 24-hour UFC is recommended with an average of at least two 24-hour urine collections due to high intra-patient variability of cortisol secretion from day to day (11, 38, 54). UFC can be influenced by sex, body mass index, age, urinary volume, and sodium intake (55-59). Urine volume and glomerular filtration rate strongly impact UFC; therefore, LNSC is preferred over UFC in patients with renal impairment (creatinine clearance <60 mL/min) or clinically significant polyuria (>5 L/24 hours) (38, 60). UFC is unable to differentiate between CS and non-neoplastic sources of hypercortisolism, which can lead to false-positive results (38, 61, 62). Additionally, there is a risk of improper sample collection, which can result in incomplete data; therefore, simultaneous measurement of urine creatinine and urine volume are necessary to verify collection completeness (63).

### Late-night salivary cortisol

LNSC assesses disruption of the normal cortisol circadian rhythm in patients with CS, specifically by detecting the loss of the physiological nighttime nadir (11, 38, 64). At least 2 LNSC samples should be collected, ideally at the patient's usual bedtime (11, 38). However, LNSC is not useful for patients with disturbed normal day and night cycles like night-shift workers (11, 38, 65, 66). Older age, uncontrolled diabetes, and other causes of non-neoplastic hypercortisolism can cause false-positives (11, 38, 67, 68). False-positive LNSC is also seen in patients who smoke, use chewing tobacco, or eat licorice (due to 11β-hydroxysteroid dehydrogenase type 2 [11β-HSD2] inhibition), or in those with direct contamination of the saliva with exogenous steroids or blood (eg, toothbrushing, gingivitis) (11, 38, 69, 70). Liquid chromatography–tandem mass spectrometry (LC-MS/MS) provides structurally specific cortisol measurements, reducing false-positives and negatives by distinguishing cortisol from cortisone and related compounds (11, 38, 71). In contrast, immunoassays are less specific due to cross-reactivity with cortisol metabolites and synthetic glucocorticoids, a limitation that applies to any cortisol measurement using an immunoassay, underscoring the importance of knowing each assay's technical specifications.

### DST

The overnight 1-mg DST assesses glucocorticoid feedback response after administration of a supraphysiologic dose of dexamethasone (38, 72). Dexamethasone is administered orally between 2300 hours and 2400 hours, and serum cortisol is measured between 0800 and 0900 hours the following day. A serum cortisol concentration ≤1.8 μg/dL (50 nmol/L) is indicative of a probable normal ACTH-cortisol axis, while values >1.8 μg/dL suggest dysregulated cortisol secretion (38, 72, 73). DST is more sensitive than LNSC or UFC in detecting mild cortisol secretion, including in patients with adrenal incidentalomas—adrenal tumors identified incidentally during imaging performed for unrelated reasons (74, 75). Dexamethasone omission, non-adherence with the protocol, increases in dexamethasone metabolism (caused by concomitant CYP3A4 inducers), poor dexamethasone absorption, concomitant oral contraceptives in women, pregnancy, and renal function impairment can cause false-positive results (51, 76-81). Plasma dexamethasone concentrations can be measured simultaneously with cortisol to confirm adequate dexamethasone absorption and rule out potential false positives (82, 83). Like LNSC and UFC, the 1-mg DST cannot differentiate between CS and non-neoplastic hypercortisolism (38, 61).

### Limitations of currently available methods to monitor cortisol

All 3 screening tests assess cortisol concentrations at a single point/day in time. Therefore, they may fail to detect cyclical CS during a low-cortisol phase (“off-cycle”) (a form of CS marked by intermittent cortisol excess) and may also yield normal or borderline results in patients with mild CS who have lower or fluctuating cortisol secretion (73, 90-93). These tests provide only a snapshot of cortisol secretion and therefore cannot capture abnormalities in its normal 24-hour rhythmic pattern. The use of multiple samples—from blood, saliva, or subcutaneous tissue—can construct a dynamic cortisol profile that offers more detailed insight into the circadian rhythm of cortisol secretion (30, 88, 94). However, this approach is considerably more invasive and inconvenient and introduces additional potential for both false-positive and false-negative results.

## Stratification of hypercortisolism

**Figure 3:**
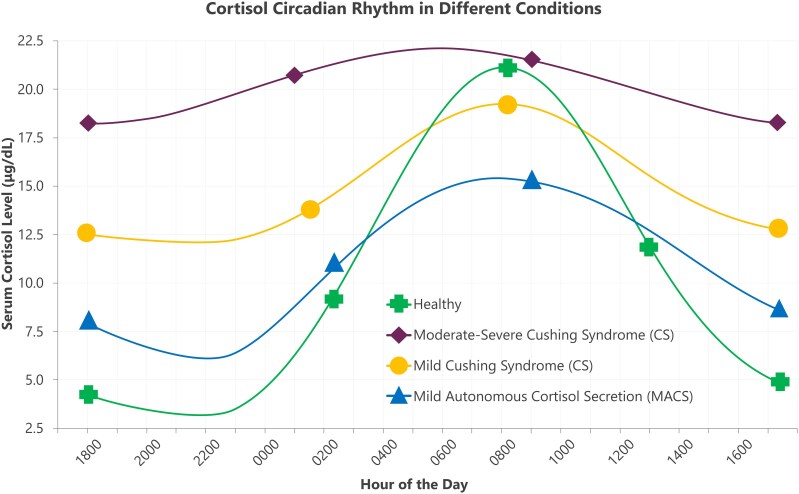
Cortisol circadian rhythm in different conditions (14, 23, 24, 29, 40, 94-96). Serum cortisol concentrations measured across a 24-hour period are shown for healthy individuals, patients with MACS, mild CS, and moderate–severe CS. Abbreviations: CS, Cushing syndrome; MACS, mild autonomous cortisol secretion.

Variability in the degree of hypercortisolism among patients contributes to a broad spectrum of clinical manifestations, ranging from MACS to severe CS (31-34). MACS refers to a condition in which unilateral or bilateral adrenal nodules/hyperplasia produce cortisol independently of usual pituitary regulation (94). Although cortisol concentrations are only mildly elevated, patients with MACS often exhibit abnormal cortisol secretion patterns—particularly elevated levels in the afternoon, evening, and nighttime—reflecting a disruption of the normal circadian rhythm (Fig. 3). A study comparing circadian secretory patterns of serum total and free cortisol in 12 patients with MACS vs 10 age- and sex-matched referent subjects observed similar 24-hour free and total serum cortisol levels but significant differences in cortisol secretion at 2200 and 0000 hours. These results emphasize the need for more reliable screening tests in patients with MACS and potentially mild CS, as commonly used biochemical tests for hypercortisolemia may lead to false-negative results.

Patients with MACS typically do not exhibit the classic clinical signs and symptoms of overt CS, characterized by clinically obvious manifestations of hypercortisolism (33). However, when evaluated using standardized clinical severity scores, certain features may overlap between MACS and CS, suggesting a continuum of cortisol excess. Although overt physical features may be absent, the subtle hypercortisolism seen in MACS has been associated with adverse cardiovascular and metabolic outcomes, including hypertension, diabetes, dyslipidemia, weight gain, osteoporosis, increased frailty, and increased mortality (33, 34, 97-101). Furthermore, the substantial overlap in both biochemical and clinical profiles between MACS and mild CS may lead to diagnostic misclassification (34).

An important factor contributing to the clinical variability observed in glucocorticoid-related conditions, such as CS and MACS, is interindividual sensitivity to glucocorticoids. This variability is influenced, in part, by polymorphisms in the glucocorticoid receptor gene, which have been associated with altered receptor function and differential metabolic responses to cortisol (102-108). As a result, patients with similar levels of cortisol exposure may exhibit different phenotypes, ranging from minimal clinical signs to overt features of cortisol excess.

Given the wide range of clinical presentations, clinical and biochemical scoring systems have recently been developed to more accurately assess the severity of hypercortisolism in patients with MACS and CS (Table 2) (33). The clinical disease severity score allows for classification of mild, moderate, or severe hypercortisolism based on the presence of metabolic abnormalities and physical features associated with excess cortisol. The biochemical disease severity score allows for severity classification of hypercortisolism based on results from biochemical screening tests (2 most abnormal tests). Patients with endogenous hypercortisolism—including both MACS and overt CS—demonstrate reduced muscle strength and impaired health-related quality of life compared to referent subjects. The study found that higher clinical severity of hypercortisolism, but not biochemical severity, was associated with worse objectively measured muscle function and lower quality of life scores, noting that the clinical severity score incorporates patient-reported proximal muscle weakness as one of its components. Patients with MACS and overt CS showed similar impairments in muscle strength and mental health, whereas patients with overt CS experienced greater physical impairment and poorer disease-specific quality of life. These findings underscore the importance of comprehensive clinical and functional assessments, alongside conventional biochemical testing, to better quantify disease burden and guide treatment across the spectrum of hypercortisolism severity.

**Table 2:** Clinical and biochemical disease severity score in patients with hypercortisolism (33)

Severity component	Parameters/scoring components	Scoring range	Median score	Clinical implications and findings
Clinical Severity Score	Assessing for metabolic and physical features associated with hypercortisolemia (HTN, abnormal glucose metabolism, decreased bone density, central obesity, facial rounding, skin changes, proximal muscle weakness, etc.)	Mild: 1 to 8 points Moderate: 9 to 14 points Severe: 15 to 22 points	CS: 15 points (IQR 11-18) Ectopic CS: 17 points (IQR 16-19) MACS: 7 points (IQR 3-10)	Reflects the burden of clinical manifestations of hypercortisolism; can differentiate between different subtypes of CS and MACS; higher scores correlated with worse QoL measures and proximal myopathy
Biochemical Severity Score	Lab measures quantifying cortisol excess (24-hour UFC, 1-mg DST, LNSC) and degree of ACTH-independence (ACTH, DHEA-S)	Mild: 0 to 3 points Moderate: 4 to 6 points Severe: 7 to 11 points	CS: 7 points (IQR 6-8) Ectopic CS: 9 points (IQR 7-11) MACS: 3 points (IQR 2-4)	Captures biochemical cortisol burden; can differentiate between different subtypes of CS and MACS; scores did not correlate with QoL measures or proximal myopathy

The table summarizes the components of the clinical severity score and biochemical severity score, including scoring components, scoring ranges, median scores for CS, ectopic CS, and MACS, and clinical implications and findings. This research was originally published in Li, et al 2023 (33)

Abbreviations: ACTH, corticotropin; CS, Cushing syndrome; DHEA-S, dehydroepiandrosterone sulfate; DST, dexamethasone suppression test; HTN, hypertension; IQR, interquartile range; LNSC, late-night salivary cortisol; MACS, mild autonomous cortisol secretion; QoL, quality of life; UFC, urinary free cortisol.

Among patients with CS, normalization of cortisol concentrations results in improvements in morbidity, mortality, and quality of life (28, 35, 109, 110). In patients with untreated hypercortisolism, both the duration and severity of hypercortisolism contribute to the development of chronic complications (28, 31-33, 46, 111, 112). Patients with CS who achieve remission following curative treatment remain at a slightly increased risk of morbidity and mortality compared to patients without CS (27, 28, 113-116). Early diagnosis is crucial, as the duration of hypercortisolism appears to be a significant factor influencing the degree of morbidity and mortality in patients with CS (46, 113). Additionally, lower baseline UFC values at the time of CS diagnosis have been associated with a higher number of long-term comorbidities, compared to patients with high UFC values at diagnosis (35). This correlation can be attributed, in part, to a longer cumulative exposure to excess glucocorticoids resulting from an insidious disorder with a delayed diagnosis. This highlights the potential value of alternative approaches to detecting and monitoring cortisol excess, particularly in patients with mild CS or MACS. There is an unmet need for minimally invasive, reliable tests to measure patients’ cortisol circadian rhythm to aid in the diagnosis and treatment monitoring of CS or MACS. Recent advances and applications are described below.

## Recent advances in cortisol measurement

### Continuous subcutaneous cortisol measurement

Continuous subcutaneous measurement of free cortisol with a portable collection device was first evaluated in 16 healthy male individuals (29). Investigators successfully reported a consecutive 72-hour free cortisol measurement depicting the characteristic cortisol circadian profile. A microdialysis catheter inserted subcutaneously allowed for ambulatory sample collection in individuals who were free to perform their day-to-day activities. A subsequent microdialysis catheter (U-RHYTHM) was developed to quantify free unbound subcutaneous concentrations of adrenal steroids, enabling characterization of 24-hour dynamic hormonal profiles in 214 healthy volunteers in an ambulatory setting (30). Subcutaneous tissue concentrations of cortisone, corticosterone, 18-hydroxycortisol, aldosterone, tetrahydrocortisol, and allo-tetrahydrocortisol were shown to coordinate with the rest–activity cycle similarly to cortisol.

Compared with current reliance on UFC, LNSC, and DST, continuous 24-hour cortisol monitoring has the potential to benefit any patient with hypercortisolism, including patients with MACS and all etiologies of CS, by providing a more accurate assessment of circadian cortisol abnormalities and nocturnal hypercortisolism, particularly in mild or borderline cases. By capturing dynamic cortisol profiles, this approach may enhance diagnostic accuracy and monitoring of treatment response while reducing stress-related false positives and improving detection of low levels of excess cortisol in mild CS or MACS that can be missed with traditional testing techniques (29, 30). However, continuous subcutaneous monitoring devices are not yet approved for clinical use, remain limited to research settings, and currently lack sufficient evidence to support their use for diagnostic purposes.

### Hair cortisol and cortisone measurement to assess long-term cortisol exposure

Hair cortisol and cortisone measurement offers a non-invasive, emerging method of assessing long-term cortisol exposure (117-119). It may be useful in detecting cyclical or mild CS by reflecting cumulative cortisol levels over weeks to months, though further studies are needed to clarify its clinical utility. The slow growth rate of hair enables the detection of long-term exposure to glucocorticoids with the ability to approximate hypercortisolism onset and degree of exposure over time. Hair analysis (typically 3 cm closest to the scalp) provides a stable measure of cortisol, reducing false-positive results from acute stressors associated with serum, saliva, or urine testing, and it can be performed at any time of the day in an outpatient setting (120). Hair analysis has also been correlated with the reduction of cortisol after medical or surgical treatment of CS (118). Prior to clinical implementation, further studies need to determine the overlap in the hair cortisol and cortisone measurements of patients with non-neoplastic hypercortisolism, MACS, and CS.

Studies evaluating hair cortisol and cortisone have reported diagnostic cutoffs ranging from approximately 5-31 pg/mg for hair cortisol and 14-28 pg/mg for hair cortisone, with sensitivities of ∼60-100% and specificities of ∼79-99%, depending on assay methodology, study population, and disease severity (Table 3) (119-121). Overall, hair cortisol and cortisone measurements can distinguish CS from controls, but substantial variability in reported cutoffs and diagnostic performance underscores the lack of validated thresholds and the need for further standardization before routine clinical use.

**Table 3:** Diagnostic performance of hair cortisol and cortisone by assay and disease severity (119-121)

Clinical scenario	Analyte	Cutoff (pg/mg)	Sensitivity	Specificity	Study
LC-MS/MS					
Overt CS vs controls	Hair cortisol	18.1	92.0%	91.3%	Brossaud et al 2021 (120)
	Hair cortisone	28.2	100.0%	98.8%	
Mild CS vs controls	Hair cortisol	12.3	59.1%	78.8%	Brossaud et al 2021 (120)
	Hair cortisone	21.7	68.2%	94.0%	
Untreated CS vs population controls	Hair cortisol	4.7	81%	88%	Savas et al 2019 (121)
	Hair cortisone	13.8	87%	90%	
ELISA					
Confirmed CS vs controls	Hair cortisol	31.1	93%	90%	Wester et al 2017 (119)

The table summarizes published cutoff values for hair cortisol and hair cortisone concentrations and their corresponding sensitivities and specificities for distinguishing patients with CS from control populations across different clinical scenarios. Cutoffs are assay- and population-specific and are not currently validated for clinical diagnosis. Cortisone generally outperforms cortisol, particularly in mild disease.

Abbreviations: CS, Cushing syndrome; ELISA, enzyme-linked immunosorbent assay; LC-MS/MS, liquid chromatography-tandem mass spectrometry.

### Salivary cortisol and cortisone to assess circadian cortisol patterns

Patients with mild CS or MACS may present with normal total 24-hour urinary cortisol production but have a disrupted cortisol circadian rhythm, often characterized by excess cortisol secretion during bedtime hours, as detected by an uncontrolled LNSC measurement (94). Detecting such subtle abnormalities through frequent blood sampling is unfeasible in this population, as samples must be collected in a clinic or hospital setting, making the process burdensome and disruptive for patients (122). LNSC has been investigated as a tool for detecting hypercortisolism in patients with mild CS or MACS; however, its sensitivity is limited in this setting, as it may fail to identify subtle disturbances in the normal cortisol rhythm (92, 93). To address these limitations, several studies have evaluated the utility of obtaining multiple salivary cortisol or cortisone measurements throughout the day to more accurately characterize cortisol circadian patterns in patients with CS or MACS (39-41, 43, 88, 122, 123). Braun et al evaluated multiple salivary cortisol measurements collected at 5 time points throughout the day to characterize daily cortisol profiles in patients with CS and MACS to assess the diagnostic utility of this approach (88). The 5-point salivary cortisol profile, comprising serial samples obtained across daytime and evening (0800, 1200, 1600, 2000, and 2300 hours) to reflect the cortisol circadian rhythm, improved diagnostic performance compared with a single late-night salivary cortisol measurement.

Salivary cortisol generally correlates well with total and free serum cortisol, while salivary cortisone shows an even stronger correlation (40). Debono et al assessed evening and nocturnal cortisol exposure in 6 patients with MACS before and after treatment with timed evening doses of metyrapone. Hourly serum cortisol, salivary cortisol, and salivary cortisone were measured during waking hours. Salivary cortisone had the strongest correlation to serum cortisol and the authors concluded that salivary cortisone may be useful for monitoring medical therapy in the community setting. Harrison et al evaluated salivary cortisone as a surrogate for 24-hour cortisol exposure and determined the minimum salivary cortisone sampling frequency needed for accurate assessment (122). In a study of 50 participants, including healthy volunteers and patients with MACS, they found that 3 salivary cortisone samples collected at 8-hour intervals reliably estimated 24-hour serum cortisol exposure. The authors emphasize the advantages of salivary measurements, noting that they are non-invasive, can be collected with minimal stress in home or workplace settings, and are highly stable. When measured by LC-MS/MS, salivary cortisone can be used in place of salivary cortisol for late-night screening for CS, particularly in situations where contamination from exogenous glucocorticoids is a concern (122, 124). As with salivary cortisol, results should be interpreted using assay-specific cutoffs and in the context of the overall clinical picture.

## Utility of cortisol in monitoring for treatment response

The goal of treating patients with CS is to normalize cortisol levels and manage associated comorbidities (37). Fully controlled CS is defined as the normalization of cortisol levels or action at its receptors in conjunction with the normalization of comorbidities by treatment of the underlying cause or treatment with adjunctive therapies. In patients with CS, surgical resection of the causative lesion(s) is the first-line treatment option regardless of etiology. Following surgical resection, postoperative serum cortisol should be measured to assess for persistent hypercortisolism, hypocortisolism, or eucortisolism (a state of normal cortisol production and regulation) (37, 38). The guidelines provide a general definition of remission after resection of an ACTH-producing corticotroph tumor as morning serum cortisol levels <5 μg/dL (<138 nmol/L) or UFC <28-56 nmol/d (<10-20 μg/d) within 7 days of tumor resection (37). More recent evidence has identified that a lower postoperative serum cortisol nadir (<2 µg/dL) is associated with long-term remission and delayed recurrence (125, 126). In patients with mild or cyclic CD, or those treated with medical therapy prior to surgery, postoperative UFC and morning cortisol may be normal (37). Late-night serum or salivary cortisol should be measured in such cases to ensure remission.

Second-line treatments should be individualized to each patient, and depending on CS subtype may include medical therapies, radiation therapy, repeat transsphenoidal surgery, or bilateral adrenalectomy (37, 38, 127). Available medical therapies currently used in clinical practice to treat CS include medications from 3 classes: pituitary-directed therapies (for pituitary CD only), steroidogenesis inhibitors, and glucocorticoid receptor antagonists (37). Pituitary-directed therapies, including pasireotide (US/EU/Canada) and cabergoline (off-label use only), inhibit pituitary ACTH production and, in some cases, ectopic ACTH secretion from neuroendocrine tumors, thereby reducing downstream cortisol secretion (37, 128). Steroidogenesis inhibitors work by inhibiting one or more enzymes involved in steroidogenesis, decreasing cortisol and other steroid hormone production (37, 38). These inhibitors include ketoconazole (EU; off-label in the US/Canada), levoketoconazole (US), metyrapone (EU; off-label in the US/Canada), osilodrostat (US/EU/Canada), mitotane (US/EU/Canada), and etomidate (off-label use only). Mifepristone (US), the only currently available glucocorticoid receptor antagonist, blocks the activation of the glucocorticoid receptor, mitigating the systemic effects of cortisol.

Medical therapy rarely induces remission; instead, the goal is to achieve disease control using cortisol levels as a surrogate marker of treatment success (37, 38). In clinical trials, the efficacy of steroidogenesis inhibitors and pituitary-directed therapies in patients with CS was primarily evaluated by the normalization of cortisol secretion per UFC (129-137). However, when glucocorticoid receptor antagonists are used, improvements in clinical signs, symptoms, and CS-induced comorbidities serve as an indicator of treatment efficiency, since biochemical measures are not reliable (37, 38). In practice, current clinical practice guidelines recommend that treatment response be assessed using a combination of clinical (eg, improved phenotype, weight, hypertension, glucose metabolism, quality of life) and biochemical endpoints.

Glucocorticoid withdrawal syndrome results from the tapering or abrupt cessation of excessive endogenous or exogenous sources of glucocorticoids (138, 139). Adrenal insufficiency is the inability of the adrenal cortex to synthesize and produce sufficient amount of glucocorticoids, mineralocorticoids, or both (140). Adrenal insufficiency and glucocorticoid withdrawal syndrome share similar clinical features and require investigations to differentiate in clinical practice (141). In glucocorticoid withdrawal syndrome, cortisol levels are typically within the normal range, whereas in adrenal insufficiency they are inappropriately low. While there is no laboratory test to diagnose glucocorticoid withdrawal syndrome, cortisol measurement provides utility in the diagnosis of adrenal insufficiency (38). UFC is not useful if adrenal insufficiency is a concern and morning serum cortisol is preferred. Low 0800 serum cortisol can aid in the diagnosis of adrenal insufficiency (138).

## Improvement of cortisol circadian rhythm after medical therapy

In patients with CS, the possibility and impact of restoring a normal cortisol circadian rhythm on morbidity, mortality, and quality of life remains unclear, highlighting a critical area for future research (20, 38). Apparent restoration of the normal cortisol circadian rhythm has been reported following surgical therapy (44). However, a more recent study found that although cortisol circadian rhythm was improved in patients in remission after surgical therapy compared to those with active disease, it was not fully restored (20). This persistent disruption in cortisol circadian rhythm during remission may play a role in the long-term complications associated with prior glucocorticoid excess (28, 113, 114, 116).

The restoration of cortisol circadian rhythm following medical therapy has not been investigated or established for most medications used to treat CS, and it is not routinely measured to determine treatment response (129-137). However, studies in patients with hypercortisolism (due to CS or MACS) show promising results (Table 4) (39-43, 110, 142).

**Table 4:** Studies reporting impact of cortisol rhythm restoration on clinical outcomes (39-43, 110, 142)

Study	Population	Intervention	Outcome measured	Key findings
van der Pas et al 2013 (39)	Patients with CD	Stepwise treatment with pasereotide, cabergoline, and ketoconazole	UFC, multiple plasma and salivary cortisol levels, and QoL-related parameters	Cortisol circadian rhythm restored in 50% of patients without correlation to QoL; QoL did not improve
Debono et al 2017 (40)	Patients with MACS	Metyrapone timed evening doses	Hourly serum and salivary cortisol levels, IL-6 (CVD risk marker)	Restoration of cortisol circadian rhythm; reduction in IL-6
Yoshida et al 2018 (41)	Patients with CS	Metyrapone	Morning serum cortisol, UFC, multiple salivary cortisol levels	UFC normalized; multiple salivary cortisol levels showed persistent cortisol circadian rhythm impairment
Newell-Price et al 2020 (42)	Patients with CD	Long-acting pasireotide	UFC, LNSC, morning serum cortisol, HRQoL, blood pressure, weight	Simultaneous control of LNSC and UFC led to greater improvements in weight and blood pressure
Newell-Price et al 2025 (110)	Patients with CD	Osilodrostat	UFC, LNSC, morning serum cortisol, CV and metabolic-related parameters, QoL	Simultaneous control of LNSC and UFC led to greater improvements in blood pressure, weight, and glucose
Ferrari et al 2025 (43)	Patients with well-controlled CS	Transition from BID osilodrostat to QD osilodrostat	Multiple serum and salivary cortisol levels, UFC, cardiometabolic markers, QoL, sleep	Improved circadian cortisol profiles, QoL, and sleep
Musolino et al 2025 (142)	Patients with mild hypercortisolism in CS	Metyrapone timed evening doses	Blood pressure, glucometabolic control, salivary cortisol circadian rhythm	Improved systolic blood pressure in 40% of patients, no differences observed in salivary cortisol

The table summarizes study populations, interventions, cortisol-related outcome measures, and key clinical findings from trials evaluating the impact of cortisol rhythm restoration on clinical outcomes.

Abbreviations: BID, twice daily; CD, Cushing disease; CS, Cushing syndrome; CV, cardiovascular; CVD, cardiovascular disease; HRQoL, health-related quality of life; IL-6, interleukin-6; LNSC, late-night salivary cortisol; MACS, mild autonomous cortisol secretion; QD, once daily; QoL, quality of life; UFC, urinary free cortisol.

van der Pas et al evaluated the effects of medical therapy on cortisol diurnal rhythm and quality of life in 12 patients with CD with abnormal baseline cortisol diurnal rhythms (39). Recovery of cortisol diurnal rhythm, measured by midnight serum and salivary cortisol levels, was achieved in 50% of patients treated with pasireotide alone or in combination with cabergoline and/or ketoconazole. This study suggests that medical therapy for CS may restore cortisol circadian rhythm, but further research is required to delineate the effectiveness of each medication in normalizing cortisol rhythms. Debono et al conducted a prospective, open-label study in 6 patients with MACS investigating evening and nocturnal cortisol exposure before and after treatment with timed evening doses of metyrapone (500 mg at 1800 hours and 250 mg at 2200 hours) (40). Abnormal 24-hour cortisol circadian rhythm, measured by hourly serum and salivary cortisol levels, was successfully corrected after metyrapone treatment. This study also displayed an association between timed metyrapone treatment and reduction in interleukin-6, a cardiovascular risk marker, highlighting the need for future investigations into whether correction of the cortisol circadian rhythm can reduce morbidity and mortality. Musolino et al evaluated the effects of timed evening doses of metyrapone (250 mg at 2200 hours) on blood pressure, glucometabolic control, and salivary cortisol rhythms in 20 patients with mild hypercortisolism due to CS (142). Unlike earlier studies, no statistically significant differences were observed in salivary cortisol AUC compared to baseline, either for total AUC or for partial intervals. Yoshida et al explored the usefulness of multiple salivary cortisol measurements in optimizing the dosage and timing of metyrapone treatment for patients with CS (41). Through 6 case presentations, they demonstrated that multiple salivary cortisol measurements detected disrupted cortisol circadian rhythms in patients despite normalized UFC levels. Additionally, a prospective study in a single patient utilized multiple salivary cortisol assessments to evaluate the cortisol circadian rhythm and successfully guide adjustments in metyrapone dose and frequency. This method of measuring the cortisol circadian rhythm offers a non-invasive tool to monitor and adjust therapy in real time, as salivary samples can be collected in outpatient settings.

Mean UFC is routinely used in clinical practice and research to monitor disease control and treatment response in patients with CS treated with medical therapy (129-137). Screening for abnormal cortisol circadian rhythm with LNSC was seldomly used to measure treatment response to medical therapy in clinical trial settings. Two separate analyses have investigated the utility of the restoration of normal cortisol circadian rhythm measured by LNSC with promising results (42, 110). Newell-Price et al conducted a pre-specified exploratory analysis evaluating LNSC levels during a Phase 3 study of long-acting pasireotide in 137 patients with CD (42). Over the 12-month treatment period, reductions in LNSC levels corresponded to changes in mean UFC. Improvements in systolic and diastolic blood pressure and weight were the greatest among patients who achieved normalization of both LNSC and UFC levels. A subsequent pooled analysis of 160 patients with CD treated with osilodrostat in 2 Phase 3 studies, assessed the possible advantages of normalizing both LNSC and mean UFC to improve treatment outcomes (110). A total of 48.6% of patients had achieved control of both LNSC and mean UFC. Investigators reported that simultaneous control of both mean UFC and LNSC resulted in generally greater improvements from baseline in cardiovascular and metabolic-related parameters of systolic blood pressure, diastolic blood pressure, fasting plasma glucose, and weight in comparison to control of only mean UFC or both LNSC and mean UFC uncontrolled. Data from these analyses support that improved regulation of late-night cortisol secretion, a key component of circadian cortisol control, as measured by UFC and LNSC, has the potential to further reduce morbidity and mortality associated with hypercortisolemia.

Ferrari et al conducted a prospective multicenter trial in 16 patients with well-controlled CS comparing the cortisol circadian rhythms between once-daily and twice-daily osilodrostat dosing regimens (43). Well-controlled disease was defined as UFC levels below the upper limit of normal for at least 1 month while on a stable twice-daily osilodrostat regimen. To assess cortisol circadian rhythms, serum and salivary cortisol and cortisone levels were measured at multiple time points over 24 hours, along with UFC while patients were on a twice-daily osilodrostat regimen (0700 and 1900 ± 1 hour) and again 60 to 90 days after transitioning to a single equivalent daily dosing (1900 ± 1 hour). Once-daily osilodrostat dosing significantly reduced late afternoon to early morning cortisol exposure while maintaining morning peak concentrations, thereby improving cortisol circadian rhythms even in patients with well-controlled CS. Additionally, patient-reported outcomes of quality of life and sleep showed significant improvement. These findings support further investigations into the potential of a chronotherapy approach to normalizing cortisol rhythms in the management of CS.

The salivary cortisol curve can provide insight into cortisol secretion patterns, but its reliability for long-term monitoring of disease activity or treatment response has not been established. Additional studies are necessary to determine its clinical value.

## Conclusions and future directions

Although disruption of the normal cortisol circadian rhythm is a key biochemical abnormality in patients with CS, current monitoring and treatment approaches do not prioritize restoring this rhythm. There is a clear need for non-invasive, non-disruptive tests to assess patients’ cortisol circadian rhythm, which could aid in the diagnosis and treatment of CS and help prevent hypercortisolism-related morbidity and mortality. Currently recommended and available tests for hypercortisolism are single-time-point tests, which are unable to effectively capture changes over the course of 24 hours to observe disruptions in the normal rhythm of adrenal hormone secretion. Recent advancements in cortisol measurement tools, such as continuous subcutaneous cortisol monitoring and multiple salivary cortisol or cortisone analysis, have the potential to detect low or fluctuating cortisol secretion in patients with cyclic CS, mild CS, or MACS—individuals who might otherwise remain undiagnosed due to the limitations of current screening tools. Additionally, given that cortisol levels alone do not reliably reflect disease severity or tissue-level cortisol activity, future research should examine the usefulness of biological markers of overall normal effectiveness of glucocorticoids on tissues, such mRNA for DSIPI, DDIT4, and FKBP5 (143). Incorporating these biomarkers into clinical studies could improve the assessment of disease activity, guide personalized medical therapy and enhance understanding of the heterogeneous clinical presentations in CS and MACS.

In patients with CS, there is currently no guidance for monitoring treatment response with respect to the restoration of cortisol circadian rhythm. Although normalization can be achieved after successful surgery, further research is required to confirm the effectiveness of medical therapy in normalizing cortisol rhythms. The possibility and impact of restoring a normal cortisol circadian rhythm on morbidity, mortality, and quality of life remains unclear, highlighting a critical area for future research. The persistent disruption of the cortisol circadian rhythm during remission may contribute to long-term complications associated with prior glucocorticoid excess, suggesting the need for future studies to determine whether correcting the cortisol circadian rhythm could help reduce morbidity and mortality.

## Data Availability

Data sharing is not applicable to this article as no datasets were generated or analyzed during the current study.

## Abbreviations

ACTH: adrenocorticotropic hormone
CD: Cushing disease
CRH: corticotropin-releasing hormone
CS: Cushing syndrome
DST: dexamethasone suppression test
HPA: hypothalamic pituitary adrenal
LC-MS/MS: liquid chromatography–tandem mass spectrometry
LNSC: late-night salivary cortisol
MACS: mild autonomous cortisol secretion
UFC: urinary free cortisol
