# Correction to: “Measuring cortisol in Cushing syndrome: diagnosis, monitoring, and cortisol circadian rhythm improvement”

**DOI:** 10.1210/clinem/dgag146

**Published:** 2026-04-06

In the above-named article by Hamidi O, St-Jean M, Lacroix A, and Bancos I (*J Clin Endocrinol Metab*. 2026; doi: 10.1210/clinem/dgag095) there was information omitted in the Funding and Acknowledgment sections.

In the originally published article, the Acknowledgment section was omitted. In the corrected article, the Acknowledgment section has been included with the statement, “The authors thank Sarah-Catherine Formiller, PharmD, BCPS and Tiago Barros Silva, PharmD, PhD, of Med Communications, Inc., for medical writing and editorial assistance provided at the direction of the authors.”

In the originally published article, in the Funding section, aspects of the article’s funding was omitted. In the corrected article, at the end of the Funding section, the following statement has been included, “Medical writing and editorial assistance was funded by Recordati Rare Diseases Inc.” The Funding section now reads as, “This research was partly supported by the National Institute of Diabetes and Digestive and Kidney Diseases of the National Institutes of Health USA under award 1K23DK142038-01A1 (to O.H.). The views expressed are those of the author(s) and not necessarily those of the National Institutes of Health USA. Medical writing and editorial assistance was funded by Recordati Rare Diseases Inc.”

The article has been corrected online.

doi: 10.1210/clinem/dgag095

